# Postoperative residual neuromuscular block in a woman with severe preeclampsia treated with magnesium sulfate and nicardipine: A case report and literature review

**DOI:** 10.3389/fsurg.2023.1093969

**Published:** 2023-02-23

**Authors:** Yingcai Song, Xiaozhe Qian, Weijia Du

**Affiliations:** ^1^ Department of Anesthesiology, Shanghai First Maternity and Infant Hospital, Tongji University School of Medicine, Shanghai, China; ^2^ Department of Thoracic Surgery, Renji Hospital, School of Medicine, Shanghai Jiaotong University, Shanghai, China

**Keywords:** calcium antagonist, general anesthesia, magnesium sulfate, neuromuscular blockade, preeclampsia

## Abstract

Calcium channel blockers and magnesium sulfate are frequently used together, particularly in women with underlying chronic hypertension and pre-eclampsia. A review of the literature showed prolonged neuromuscular blockade after treatment with magnesium sulfate. Since magnesium and calcium have opposite effects on the neuromuscular junctions, muscle weakness may become a postoperative manifestation of magnesium sulfate and calcium antagonist treatment in the obstetric population; however, limited information is available regarding this postulation. Here, we report a case wherein rocuronium activity was markedly prolonged due to pretreatment with magnesium sulfate and nicardipine after general anesthesia during an emergency cesarean delivery.

## Introduction

Pre-eclampsia is a relatively common complication of pregnancy. Magnesium sulfate is recommended for the prevention and treatment of eclamptic seizures, and calcium antagonists are used to control pregnancy-related hypertension. The combination of both these agents may have severe consequences (1). Contemporaneous administration of these drugs resulted in reversible and transient neuromuscular blockade in two cases (2, 3), and caused only mild neuromuscular weakness and blockade in another retrospective study (4). Magnesium sulfate, when administered prior to induction, increased neuromuscular blockade duration by 25% (5) and delayed restoration of consciousness in the non-obstetric population (6). Nicardipine, a calcium channel blocker, also potentiated nondepolarizing muscle relaxants *in vitro* (1) and may theoretically produce similar clinical effects. However, limited data is available regarding the interaction between these drugs and the resultant neuromuscular blockade in obstetric populations. Here, we report prolonged rocuronium nondepolarizing activity due to magnesium sulfate and nicardipine in a parturient.

## Case report

A 32-year-old woman (weight, 70 kg; gravida 1, para 0) with an uneventful antenatal course until 29 weeks of gestation, presented with a high blood pressure of 170/110 mmHg and urine protein level of 4+ on admission. She complained of headache and swelling of the feet and legs 2 weeks prior to admission. She had a history of diabetes mellitus before pregnancy. The uterine fundal height was lower than expected. Examinations revealed the presence of symmetrical peripheral edema and a pleural effusion. Blood tests showed a platelet count of 55 × 10^9^/L, elevated levels of lactic dehydrogenase (422 U/L) and uric acid (450 µmol/L). On ultrasound assessment, the baby's growth corresponded to the 3.6th percentile, and the circumferences of the head and abdomen corresponded to the 0.8th and 3.4th percentiles for gestational age, respectively. Severe preeclampsia and fetal growth restriction were diagnosed. The patient was administered 200 mg labetalol orally for hypertension, and a loading dose of 4 g magnesium sulfate intravenously for seizure prophylaxis. The blood pressure remained high; furthermore, the platelet count dropped to 50 × 10^9^/L. An infusion of 3 mg/h nicardipine was initiated. Due to continuing symptoms of headache, high blood pressure (>160/110 mmHg), and progressively decreased platelet count, decision of emergency delivery was made and was undertaken by cesarean section. General anesthesia was induced with propofol and rocuronium 30 mg (0.43 mg/kg), and the trachea was intubated. Anesthesia was maintained using sevoflurane 2.0%–2.5% until delivery. After delivery, 2 mg midazolam, 30 µg sufentanil, and 20 mg rocuronium were injected along with continuous infusion of propofol (100 mcg/kg/min).

The duration of the surgery was about 1 h. After the surgery, patients were removed to post-anesthesia care unit with endotracheal tubes. Blood pressure was maintained between 140/90 and 150/100 mmHg with continuous nicardipine infusion (5 mg/h), followed by a platelet transfusion. One hour later, the patient had no spontaneous breathing and was unresponsive to stimulation. Her body temperature was 36.3°C, and urine output was 60 ml/h. Arterial blood gas measurements showed a therapeutic level of magnesium, with hyperkalemia and hypocalcemia (Mg^2+^, 2.5 mmol/L; K^+^, 6.1 mmol/L; Ca^2+^, 1.11 mmol/L). The patient was administered 1 g calcium gluconate. Quantitative train of four (TOF) monitoring demonstrated 0 of 4 twitches, and 200 mg sugammadex was administered intravenously. The TOF ratio was measured at >90% 10 min after sugammadex administration. The coughing and swallowing reflexes returned. The patient was extubated uneventfully and transferred to the intensive care unit.

Seven hours postdelivery, intravenous infusion of 15 g magnesium sulfate (1.5 g/h) was started. On the postoperative day (POD), the serum creatinine and uric acid levels increased significantly with a decline in the glomerular filtration rate (GFR), and renal function. Brain computed tomography findings were normal; thereby excluding neurological complications of preeclampsia. On POD4, liver enzyme levels began to increase. Hemolysis was not observed. Her serum magnesium levels remained high for some days. Within a week, the blood profile, and renal and liver functions normalized and the patient and baby were discharged. Written informed consent was obtained from the patient for the publication of this case report. A copy of the written consent form is available for review.

## Discussion

Magnesium sulfate and nicardipine are used for preeclampsia treatment. We conducted a literature review of studies on obstetric populations simultaneously treated with magnesium sulfate, calcium antagonists, and neuromuscular blockers (Table 1). Feng et al. (7) reported that a woman with severe preeclampsia had a high serum magnesium level (4.11 mmol/L) with severe metabolic and respiratory acidosis, possibly causing delayed recovery. Many times, magnesium sulfate infusion was continued until anesthesia induction (Table 1). However, here, magnesium sulfate was stopped after the loading dose since nicardipine was administered. Here, the concomitant use of magnesium sulfate and a calcium antagonist was not recommended due to the potential risk of severe maternal hypotension (15, 16). Magnesium sulfate was stopped for over 4 h, and serum magnesium was maintained in the low therapeutic range. The parturient showed adequate urinary output without acidosis. Therefore, the possibility of magnesium toxicity was excluded. Moreover, increased serum creatinine and uric acid levels indicated acute renal function decline, implying a major role in sustained higher serum magnesium levels. Inadequate renal function could alter rocuronium's pharmacokinetics and magnesium secretion, causing prolonged blockade (17), thereby delaying recovery.

**Table 1:** Findings in case reports describing the interactions among MgSO_4_, calcium antagonists, and neuromuscular blockers in the obstetric population.

Case report	Relevant neuromuscular blocker	Characteristics of MgSO_4_ administration	Serum concentration of Mg	Characteristics of calcium antagonist administration	Clinical symptoms
Feng et al. (7)	Rocuronium 40 mg (0.55 mg/kg)	MgSO_4_ 14 g was infused for 2 h before induction.	4.11 mmol/L	Not mentioned	Retained unconsciousness for more than 1 h after the surgery.
Skaredoff et al. (8)	Succinylcholine 100 mg	MgSO_4_ was infused at 1 g/h after a 4 g loading dose.	4.0 mmol/L	Not mentioned	Resumed spontaneous breathing 4 h 20 min after succinylcholine administration.
Ghoneim et al. (9)	d-Tubocurarine 24 mg	MgSO_4_ 10 g IM was administered before the symptom occurred.	8.4 mg/ml (normal, 1.2–2.9 mg/ml)	Not mentioned	Difficulty in breathing and swallowing, tendon reflexes disappeared after 10 g MgSO_4_ IM injection.
	d-Tubocurarine 30 mg	Last dose of MgSO_4_ 10 g IM was administered 3.5 h before induction.	Not reported	Not mentioned	Manually ventilated for more than 5 h after the surgery.
Sinatra et al. (10)	Vecuronium 3 mg (0.05 mg/kg)	MgSO_4_ 4 g IV bolus was administered twice followed by a 2 g/h continuous infusion.	6.2 mEq/L	Not mentioned	Extubated 3.5 h after receiving a single dose of vecuronium.
Berdai et al. (11)	Vecuronium 6 mg (0.1 mg/kg)	MgSO_4_ was IV administered with a 4 g loading dose and continued at 1 g/h.	Not reported	Nicardipine 2 mg/h was administered preoperatively	Extubated 4 h after the surgery using neostigmine.
Yoshida et al. (12)	Vecuronium 3 mg (0.05 mg/kg)	MgSO_4_ was continuously infused before the cesarean section.	4.5 mg/dl	Not mentioned	Muscle weakness and re-intubation 50 min after extubation; ventilated in the following 5 h.
	Vecuronium 2.5 mg (0.05 mg/kg)	Continuous IV MgSO_4_ retained the plasma concentration of magnesium at 5–6 mg/dl.	4.8 mg/dl	Not mentioned	Extubated 5 h after vecuronium injection.
Hino et al. (13)	Vecuronium 5.8 mg (0.1 mg/kg)	IV MgSO_4_ 2 g/day for 10 days preoperatively.	3.3 mEq/L	Not mentioned	Prolonged duration of vecuronium (75 min).
Moriwaki et al. (14)	Rocuronium 40 mg (0.94 mg/kg)	MgSO_4_ was infused at 1 g/h after loading infusion at 4 g/20 min until anesthesia induction.	4.4 mg/dl	Nifedipine 20 mg was administered orally 5 h before anesthesia induction.	Extubated 3 h after rocuronium injection with sugammadex and neostigmine.

IM, intramuscularly; IV, intravenously; MgSO_4_, magnesium sulfate.

Magnesium sulfate enhances the effect of neuromuscular blockers (18). Skaredoff et al. (8) reported prolonged succinylcholine depolarizing activity due to high magnesium levels. Additionally, nondepolarizing neuromuscular agents can also interact with magnesium sulfate. Ghoneim et al. (9) reported magnesium sulfate -mediated potentiation of neuromuscular blockade of tubocurarine (9). Hino et al. (13) reported rapid onset and long duration of vecuronium in a parturient pretreated with magnesium sulfate. Similarly, Berdai et al. (11) reported that elevated liver enzymes and low platelet count syndrome predisposed to prolonged neuromuscular blockade. Yoshida et al. (12) and Sinatra et al. (10) reported prolonged vecuronium action induced by magnesium sulfate after general anesthesia despite vecuronium dose reduction. Moriwaki et al. (19) presented a case similar to ours wherein the parturient underwent reversal with sugammadex and neostigmine 3 h after rocuronium injection. However, magnesium sulfate infusion was maintained until 10 min before anesthesia induction in that case.

Similarly to calcium, magnesium regulates muscle contraction and neuromuscular excitability. Increased magnesium concentrations interfere with calcium binding to vesicles, preventing acetylcholine release at neuromuscular junctions (14). This inhibits end-plate depolarization and depresses muscle fiber excitability (9). However, neuromuscular function can be improved with high calcium levels. In a case by Ghoneim et al. (9), the parturient received 60 g magnesium sulfate over 15 h, and the reversal was inadequate for extubation, but this was attempted with a single calcium gluconate (1 g) dose. Rocuronium 0.9–1.2 mg/kg shows a duration of approximately 60 min (5). Our patient received rocuronium 0.74 mg/kg for over 4.5 h after the last magnesium dose; however, the time to achieve 90% TOF was over 2 h despite the administration of 1 g calcium gluconate. Therefore, it may be speculated that the calcium dose was inadequate.

Calcium antagonists potentiate magnesium toxicity and enhance the effects of muscle relaxants (3). A case by Ben-Ami et al. (2) suggested that serum magnesium in combination with nifedipine could cause neuromuscular blockade. In a case by Berdai et al. (11), magnesium sulfate and nicardipine caused prolonged and deep neuromuscular blockade in a preeclampsia patient. In contrast, a study with most women (96%) receiving short-acting nifedipine capsules (5-mg dose) reported that simultaneous administration of magnesium sulfate and nifedipine did not increase the risk of neuromuscular weakness (4). Here, however, nicardipine infusion was maintained preoperatively and during the recovery period.

This case reports the possibility of prolonged rocuronium nondepolarizing activity due to pretreatment with magnesium sulfate and a calcium antagonist. It illustrates the importance of using reduced-dose rocuronium, performing adequate neuromuscular monitoring, and informing the parturients of the possibility of delayed extubation and postoperative respiratory support on treatment or pretreatment with both magnesium sulfate and calcium antagonists.

## Data Availability

The original contributions presented in the study are included in the article/Supplementary Material, further inquiries can be directed to the corresponding author.
